# Proteomic profiling of impaired excitation–contraction coupling and abnormal calcium handling in muscular dystrophy

**DOI:** 10.1002/pmic.202200003

**Published:** 2022-08-08

**Authors:** Paul Dowling, Stephen Gargan, Dieter Swandulla, Kay Ohlendieck

**Affiliations:** ^1^ Department of Biology, Maynooth University National University of Ireland Maynooth, Co. Kildare Ireland; ^2^ Kathleen Lonsdale Institute for Human Health Research Maynooth University Maynooth, Co. Kildare Ireland; ^3^ Institute of Physiology University of Bonn Bonn Germany

**Keywords:** calcium homeostasis, dihydropyridine receptor, dystrophin, dystrophinopathy, ryanodine receptor, triad

## Abstract

The X‐linked inherited neuromuscular disorder Duchenne muscular dystrophy is characterised by primary abnormalities in the membrane cytoskeletal component dystrophin. The almost complete absence of the Dp427‐M isoform of dystrophin in skeletal muscles renders contractile fibres more susceptible to progressive degeneration and a leaky sarcolemma membrane. This in turn results in abnormal calcium homeostasis, enhanced proteolysis and impaired excitation–contraction coupling. Biochemical and mass spectrometry‐based proteomic studies of both patient biopsy specimens and genetic animal models of dystrophinopathy have demonstrated significant changes in the concentration and/or physiological function of essential calcium‐regulatory proteins in dystrophin‐lacking voluntary muscles. Abnormalities include dystrophinopathy‐associated changes in voltage sensing receptors, calcium release channels, calcium pumps and calcium binding proteins. This review article provides an overview of the importance of the sarcolemmal dystrophin‐glycoprotein complex and the wider dystrophin complexome in skeletal muscle and its linkage to depolarisation‐induced calcium‐release mechanisms and the excitation–contraction–relaxation cycle. Besides chronic inflammation, fat substitution and reactive myofibrosis, a major pathobiochemical hallmark of X‐linked muscular dystrophy is represented by the chronic influx of calcium ions through the damaged plasmalemma in conjunction with abnormal intracellular calcium fluxes and buffering. Impaired calcium handling proteins should therefore be included in an improved biomarker signature of Duchenne muscular dystrophy.

**Abbreviations**
AQ: aquaporin
CR: cysteine‐rich domain
DMD: Duchenne muscular dystrophy
Dp: dystrophin protein
EC: excitation contraction
ECCE: excitation‐coupled calcium entry
H: hinge region
L‐type: long‐lasting activation
NADPH: reduced nicotinamide adenine dinucleotide phosphate
nNOS: neuronal nitric oxide synthase
MG: mitsugumin
ORAI: calcium release‐activated calcium modulator
PKC: protein kinase C
SERCA: sarcoplasmic reticulum calcium ATPase
SLR: spectrin‐like region
SOCE: store‐operated calcium entry
STAC: SH3 and cysteine‐rich domain‐containing protein
STIM: stromal interaction molecule
TnC: troponin subunit‐C
TRPC: transient receptor potential channel
VDAC: voltage‐dependent anion channel

## 1 INTRODUCTION

Skeletal muscle fibres form the largest tissue mass in the human body. The skeletal musculature, consisting of over 650 individual muscles, plays a key role in voluntary movements, posture, heat homeostasis and metabolic integration [1]. Acquired or inherited abnormalities in the physiological regulation or biochemical functioning of the contractile system is therefore often associated with deleterious effects on whole body homeostasis [2, 3]. The most frequently inherited disorder of the neuromuscular system is Duchenne muscular dystrophy, which is characterised by primary abnormalities in the X‐chromosomal *DMD* gene [4] resulting in the almost complete loss of the dystrophin isoform Dp427‐M in skeletal muscle tissues [5]. Histopathological features of skeletal muscle degeneration include progressive myonecrosis that is accompanied by sterile inflammation, fat substitution and reactive myofibrosis [6]. Besides the voluntary contractile system, a variety of other organs are affected in X‐linked muscular dystrophy which can result in late‐onset cardiomyopathy, the cardio‐renal syndrome, gastrointestinal malfunctions, liver impairment and abnormal functioning of the central nervous system [7]. This multi‐systems pathophysiology and considerable organ crosstalk make dystrophinopathy a highly complex disorder that severely affects the quality of life of Duchenne patients [8] and is associated with a high caregiver burden [9].

Of central importance for the molecular pathogenesis of X‐linked muscular dystrophy is the collapse of the dystrophin‐glycoprotein complex, which supports in normal muscle the organisation of the intracellular cytoskeletal network, the linkage between the sarcolemma and the basal lamina of the extracellular matrix, lateral force transmission and cellular signalling [10]. Mass spectrometry‐based proteomics has been widely applied to study changes in dystrophic skeletal muscles linked to the loss of the dystrophin complex and associated secondary alterations in other tissues and biofluids. This analytical approach has established a comprehensive and disease‐specific biomarker signature of dystrophinopathy [11, 12, 13, 14, 15]. In general, proteomic analyses can be carried out in top‐down versus bottom‐up approaches, which utilise proteoform‐centric techniques [16] versus peptide‐focussed methodologies [17] for the identification and characterisation of individual protein species, respectively. The bioanalytical combination of large‐scale protein separation techniques, which are mostly based on sophisticated gel electrophoretic and/or liquid chromatographic methods, and a variety of sensitive mass spectrometric procedures are employed to study the complexity of proteoforms and their dynamic post‐translational modifications [18, 19, 20].

Systematic mass spectrometric surveys of both patient muscle specimens and animal models of X‐linked muscular dystrophy have identified significant proteome‐wide disturbances/adaptations in cytoskeletal networks, the extracellular matrix, the contractile apparatus, energy metabolism and the cellular stress response in contractile fibres [21, 22, 23]. These complex alterations in specific muscle‐associated proteoforms agree with the highly progressive pathogenesis of dystrophinopathy [24]. Importantly, experiments with dystrophic skeletal muscles that underwent stretching during contraction showed that more extensive damage is caused during eccentric contractions as compared to normal muscle [25, 26, 27]. In general, muscles undergoing eccentric contraction patterns are highly susceptible to tissue destruction, causing leaky surface membranes and reduced contractile force [28]. Studies with a focus on the characterisation of the cellular damage triggered by eccentric contractions in skeletal muscles from the *mdx* mouse established this spontaneous model of Duchenne muscular dystrophy as a suitable system to study stretch‐induced tissue disruptions [29, 30, 31].

**Significance Statement**

This review highlights the important contribution of abnormal calcium handling and impaired excitation–contraction coupling in Duchenne muscular dystrophy, a neuromuscular disorder that is triggered by the disintegration of the dystrophin‐glycoprotein complex. The article highlights the findings from mass spectrometry‐based proteomic studies of dystrophin‐deficient skeletal muscles and discusses the pathophysiological consequences of alterations in the abundance and function of key calcium‐regulatory proteins.

One of the major pathophysiological mechanisms that trigger progressive skeletal muscle fibre wasting in dystrophin‐deficient skeletal muscles is the dysregulation of calcium homeostasis [32, 33, 34]. The loss of dystrophin and accompanying disintegration of the dystrophin‐associated glycoprotein complex was shown to be linked to the destabilisation of the extracellular matrix‐sarcolemma‐actin cytoskeleton axis [35]. This in turn triggers micro‐rupturing of the surface membrane system and abnormal Ca^2+^‐fluxes that result in the increased proteolytic degradation of skeletal muscle proteins [36, 37, 38]. Progressive damage due to proteolysis and disturbed Ca^2+^‐handling severely influence the fine tuning of the excitation–contraction coupling process [39, 40, 41]. Since the conformational coupling between the ryanodine receptor Ca^2+^‐release channels of the sarcoplasmic reticulum and the voltage‐sensing L‐type Ca^2+^‐channels of the transverse tubules play a central role in depolarisation‐induced Ca^2+^‐release [42], receptor dysregulation and ion channel leakiness disturb the proper linkage between motor neuron activity and skeletal muscle functioning [43, 44, 45]. In addition, stretch‐induced fibre damage, Ca^2+^‐entry through mechano‐sensitive channels, altered membrane permeability and sarcosolic Ca^2+^‐overload are also associated with mitochondrial dysregulation involving the mitochondrial transition pore in dystrophic muscle fibres. Mitochondrial abnormalities lead to elevated levels of reactive oxygen species via NADPH oxidase activity and disturbed energy metabolism [46, 47, 48, 49].

In this review, we describe the pathophysiological mechanisms that underlie abnormal skeletal muscle functioning in X‐linked muscular dystrophy from a proteomics perspective. The main focus of this article is on the proteomic profiling of key proteins involved in excitation–contraction coupling and Ca^2+^‐homeostasis. This includes the description of the main players of Ca^2+^‐handling that are involved in the temporal and spatial regulation of Ca^2+^‐fluxes, Ca^2+^‐pumping, Ca^2+^‐buffering and Ca^2+^‐sensing in skeletal muscles [50, 51, 52]. This is followed by an outline of the calcium hypothesis of Duchenne muscular dystrophy and the discussion of recent findings from biochemical and proteomic analyses of abnormal Ca^2+^‐handling and disturbed cellular signalling in dystrophinopathy.

## 2 THE DYSTROPHIN‐ASSOCIATED PROTEIN COMPLEX AND MUSCULAR DYSTROPHY

**FIGURE 1:**
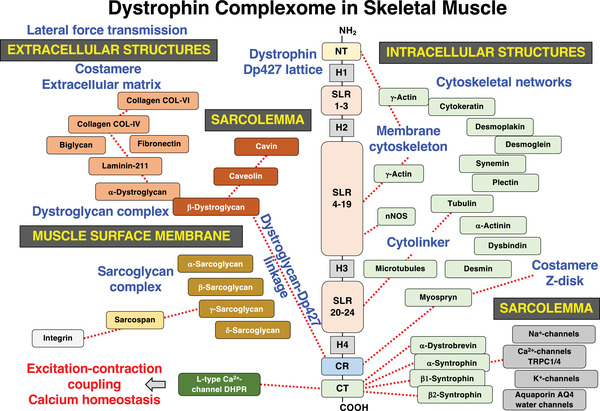
Overview of molecular domains and binding sites in the full‐length Dp427‐M isoform of skeletal muscle dystrophin, the interaction sites of members of the core dystrophin‐glycoprotein complex and its secondarily associated components of the extracellular matrix, sarcolemma and intracellular cytoskeleton. AQ, aquaporin; CT, carboxy‐terminus; CR, cysteine‐rich domain; DHPR, dihydropyridine receptor; Dp427, full‐length dystrophin protein of 427 kDa; H, hinge region; nNOS, neuronal nitric oxide synthase; NT, amino‐terminus; SLR, spectrin‐like domain; TRPC, transient receptor potential channel

The disintegration of the dystrophin‐associated protein complex is of central importance for our understanding of the pathobiology of Duchenne muscular dystrophy [5, 7]. As outlined in Figure 1, the dystrophin complexome of skeletal muscle encompasses integral proteins of the sarcolemma, components of the intracellular cytoskeletal networks and proteins of the basal lamina and the wider extracellular matrix [10, 35, 53]. The *DMD* gene contains a variety of tissue‐specific promoters that produce eight major dystrophins, that is, neuronal Dp45, ubiquitous Dp72‐G, Schwann cell Dp116‐S, brain and kidney Dp240‐B/K, retina Dp‐260‐R, brain Dp427‐B, Purkinje cell Dp427‐P and skeletal/cardiac/smooth muscle Dp427‐M [5]. In skeletal muscles, the full‐length Dp427‐M isoform of dystrophin consists of an amino‐terminal domain with a major γ‐actin binding site, 4 proline‐rich hinge regions (H1‐H4) that are distributed throughout the 427 kDa protein molecule, 3 spectrin‐like regions (SLR 1–3, SLR 4–19 and SLR 20–24) with binding sites for γ‐actin, the neuronal nitric oxide synthase nNOS and microtubules, a cysteine‐rich domain (CR) with a binding site for the integral glycoprotein β‐dystroglycan and the Z‐disc/costamere scaffolding protein myospryn, and a carboxy‐terminal domain that tightly interacts with the cytosolic proteins dystrobrevin and syntrophin [53] (Figure 1).

The core dystrophin lattice is also linked to the integral α/β/γ/δ‐sarcoglycan complex and the highly hydrophobic membrane protein sarcospan [54]. In addition, the wider assembly of Dp427‐associated proteins consists of additional sarcolemmal proteins (cavin, caveolin, integrin, Na^+^‐channels, Ca^2+^‐channels, K^+^‐channels and the water channel aquaporin AQ4), various cytoskeletal proteins (cytokeratin, plectin, tubulin, α‐actinin, dysbindin, desmin, synemin, desmoplakin and desmoglein) and extracellular matrix proteins (α‐dystroglycan, laminin‐211, biglycan, fibronectin and collagens) [10]. A potential direct linkage of the dystrophin complex to the excitation–contraction coupling apparatus has been postulated by Friedrich et al. [55], who could demonstrate that L‐type Ca^2+^‐channel function is connected to dystrophin expression in skeletal muscle tissue.

X‐linked muscular dystrophy is characterised by the almost complete loss of dystrophin isoform Dp427‐M in skeletal muscles and the concomitant collapse of the above‐described dystrophin‐associated glycoprotein complex plus various adaptations in the wider dystrophin complexome. This includes a drastic reduction in α/β‐dystroglycan, α‐dystrobrevin, α/β1/β2‐syntrophin, α/β/γ/δ‐sarcoglycan and sarcospan in contractile fibres [24, 53] and the myofibrosis‐related increase in extracellular matrix proteins such as fibronectin, periostin, proteoglycans and various collagens [56]. Importantly, disintegration of the dystrophin complex results in the loss of a proper cellular linkage between the intracellular actin membrane cytoskeleton and the extracellular basal lamina, which is otherwise provided by the sarcolemmal dystrophin–dystroglycan connection in normal contractile fibres [35]. This lack of dystrophin‐related membrane stability renders skeletal muscle fibres more susceptible to contraction‐induced sarcolemmal micro‐rupturing [27, 57, 58], which in turn results in dysregulation of ion homeostasis and forms the corner stone of the calcium hypothesis of Duchenne muscular dystrophy as described below.

## 3 EXCITATION–CONTRACTION COUPLING AND CALCIUM HOMEOSTASIS IN SKELETAL MUSCLE

**FIGURE 2:**
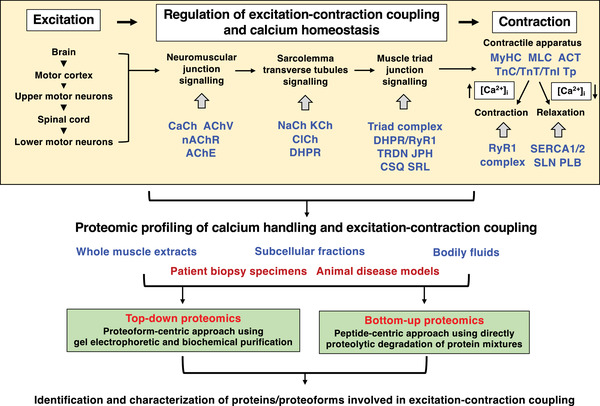
Summary of the main proteins involved in excitation–contraction coupling and an overview of proteomic approaches used to study changes in Ca^2+^‐regulatory proteoforms. AChE, acetylcholinesterase; AChV, acetylcholine vesicle; ACT, actin; CaCh, calcium channel; CSQ, calsequestrin; DHPR, dihydropyridine receptor; JPH, junctophilin; KCh, potassium channel; MLC, myosin light chain; MyHC, myosin heavy chain; NaCh, sodium channel; nAChR, nicotinic acetylcholine receptor; PLB, phospolamban; RyR1, ryanodine receptor calcium release channel; SERCA; sarcoplasmic reticulum calcium ATPase; SLN, sarcolipin; SRL, sarcalumenin; TnC, troponin subunit‐C; TnI, troponin subunit‐I; TnT, troponin subunit‐T; Tp, tropomyosin; TRDN, triadin

This review focusses on the pathophysiological role of abnormal Ca^2+^‐signalling during the well‐established process of excitation–contraction coupling [50, 51, 52]. Figure 2 summarises the main steps and proteins that are important for excitation–contraction coupling, as well as the mass spectrometry‐based proteomic approaches used to identify skeletal muscle proteins involved in Ca^2+^‐handling. Besides mechanisms of Ca^2+^‐fluxes and ion cycling in relation to orthograde excitation–contraction coupling and muscle fibre relaxation, Ca^2+^‐homeostasis in skeletal muscle tissues involves several additional processes [32], such as excitation‐coupled Ca^2+^‐entry (ECCE) [59] and store‐operated Ca^2+^‐entry (SOCE) whereby Ca^2+^‐ions derive from the extracellular space surrounding muscle fibres [60]. In this context, it should be noted that the dihydropyridine receptor has a dual function in skeletal muscles [61]. Most importantly the α1S‐subunit serves as voltage sensor in excitation–contraction coupling [50, 51, 52], but also functions as an L‐type Ca^2+^‐channel which allows ECCE [62]. Of note, multiple signalling interactions occur in a bidirectional way at the triad junction between the transverse tubular L‐type Ca^2+^‐channel and the Ca^2+^‐release channel of the sarcoplasmic reticulum [63]. On the one hand, depolarisation‐induced conformational changes in the voltage‐sensing L‐type Ca^2+^‐channel translate into opening of the Ca^2+^‐release channel in the sarcoplasmic reticulum during orthograde signalling that regulates excitation–contraction coupling [64]. On the other hand, retrograde coupling between L‐type Ca^2+^‐channels and Ca^2+^‐release channels has multiple effects on the expression and functioning of the transverse tubular L‐type Ca^2+^‐channel [65]. In addition, the chronic elevation of the sarcosolic Ca^2+^‐concentration and increased Ca^2+^‐transients in dystrophic fibres induce mitochondrial Ca^2+^‐overload and retrograde Ca^2+^‐release [32]. The pathophysiological importance of crucial aspects of these additional Ca^2+^‐regulatory processes have been extensively discussed in previous reviews [33, 34, 66].

Once the physiological unit between an α‐motor neuron and all its innervated contractile fibres is activated at the neuromuscular junction and sufficient quanta of pre‐synaptically released acetylcholine neurotransmitters diffuse through the subsynaptic cleft to the nicotinic acetylcholine receptors of the post‐synaptic junctional folds, the inward flux of Na^+^‐ions triggers depolarisation of the skeletal muscle sarcolemma. The transient opening of voltage‐dependent Na^+^‐channels and subsequent activation of delayed rectifying K^+^‐channels results in the controlled propagation of action potentials along the muscle surface membrane and deep into the muscle fibre interior via the transverse tubular system [67]. The voltage‐sensing dihydropyridine receptor of the transverse tubules, consisting of the main α1S‐subunit plus auxiliary α2/δ, β and γ subunits [68, 69], is closely linked to the Ca^2+^‐release channel of the junctional sarcoplasmic reticulum [51, 70], which in turn is directly associated with the luminal clusters of the Ca^2+^‐binding protein calsequestrin in the terminal cisternae region [71, 72]. The central regulatory step at the triad junction is provided by conformational coupling [73, 74, 75], that is, protein–protein interactions between the II–III loop domain of the α1S‐subunit of the L‐type Ca^2+^‐channel dihydropyridine receptor (Cav1.1) [67, 76] and the RyR1 isoform of the tetrameric ryanodine receptor Ca^2+^‐release channel foot structure [77, 78]. Following RyR1 activation by coupling to the voltage‐sensing dihydropyridine receptor, conformational changes in the ryanodine receptor complex result in the transient opening of an ion pore enabling the passive fluxing of Ca^2+^‐ions from the luminal sarcoplasmic reticulum into the sarcosol along a steep ion gradient [79].

The efficient provision of large quantities of luminal Ca^2+^‐ions for efflux is mediated by calsequestrin [80], the most abundant high‐capacity Ca^2+^‐binding protein of the sarcoplasmic reticulum [81]. Additional Ca^2+^‐buffering components are sarcalumenin and the histidine‐rich Ca^2+^‐binding protein [67]. Importantly, the isoform expression pattern of many Ca^2+^‐regulatory proteins differs between fast‐twitching versus slow‐twitching muscle fibres [82], making them ideal markers of muscle fibre type shifting in neuromuscular diseases [83]. A variety of minor junctional proteins are involved in triad architecture and stabilisation of the depolarisation‐induced Ca^2+^‐release mechanism including triadin, junctin, juntophilin‐1, junctophilin‐2 and mitsugumin MG‐29 [84, 85, 86, 87], as reviewed by Treves et al. [88]. Additional Ca^2+^‐regulatory elements are represented by the stromal interaction molecule STIM1, the calcium release‐activated calcium modulator ORAI1, the SH3 and cysteine‐rich domain‐containing protein STAC3 and various Ca^2+^‐leak channels [89, 90, 91], as well as intracellular organelle proteins such as the mitochondrial Na^+^/Ca^+^‐exchanger, H^+^/Ca^2+^‐exchanger, Ca^2+^‐uniporter complex and the porin VDAC (voltage‐dependent anion channel) complex [92, 93].

**FIGURE 3:**
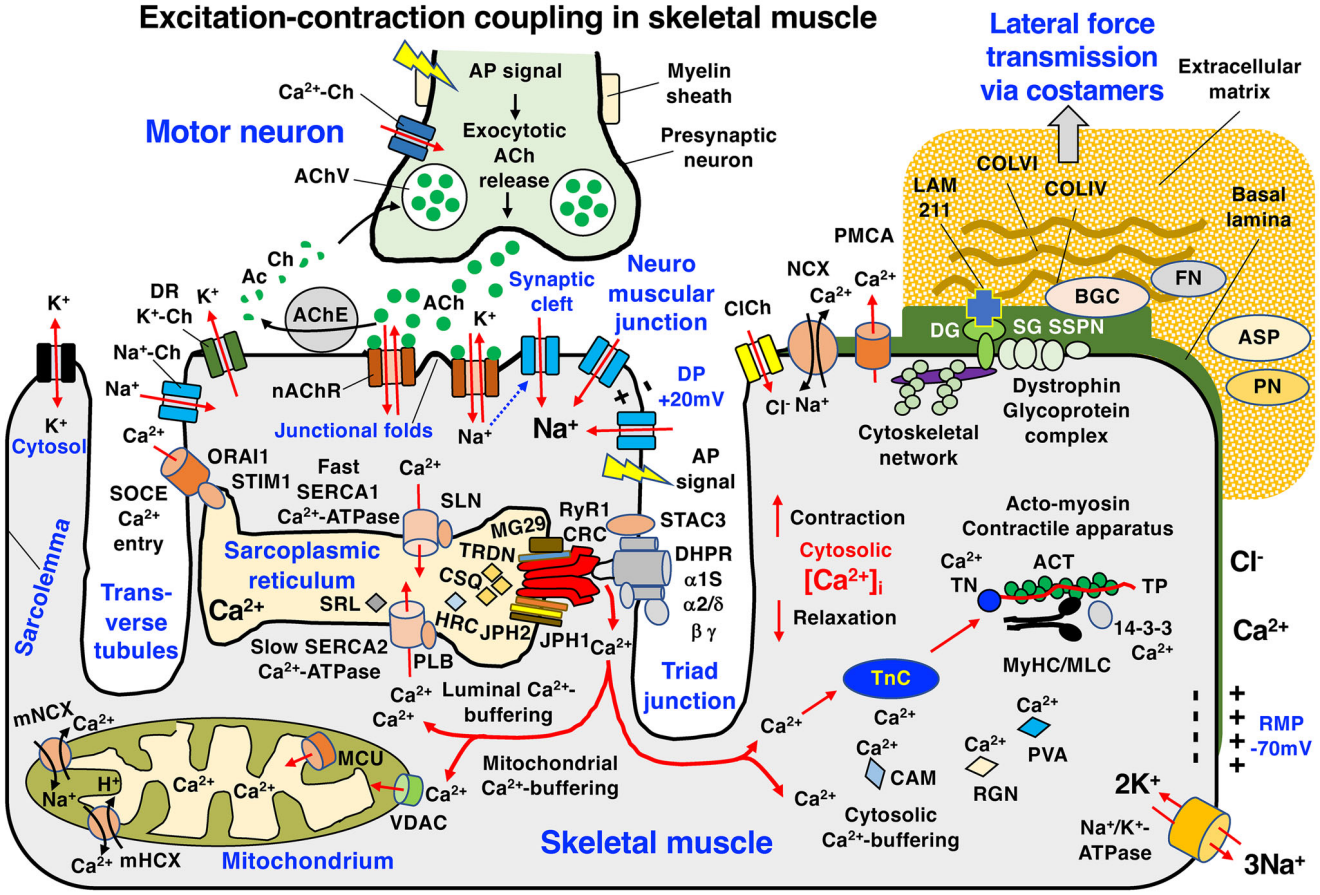
Diagrammatic overview of the complex arrangement of the proteins involved in excitation–contraction coupling and Ca^2+^‐homeostasis in skeletal muscles. ACh, acetylcholine; AChE, acetylcholinesterase; AChV, acetylcholine vesicle; ACT, actin; AP, action potential; ASP, asporin; BGC, biglycan; CaCh, calcium channel; CAM, calmodulin; ClCh, chloride channel; COL, collagen; CSQ, calsequestrin; DG, dystroglycan; DHPR, dihydropyridine receptor; DP, depolarisation; DR, delayed rectifier; FN, fibronectin; HRC, histidine‐rich calcium binding protein; JPH, junctophilin; KCh, potassium channel; LAM, laminin; MCU, mitochondrial calcium uniporter; MG29, mitsugumin‐29; MLC, myosin light chain; mHCX, mitochondrial hydrogen‐calcium exchanger; mNCX, mitochondrial sodium‐calcium exchanger; MyHC, myosin heavy chain; NaCh, sodium channel; nAChR, nicotinic acetylcholine receptor; NCX, sodium‐calcium exchanger; ORAI1, calcium release‐activated calcium modulator 1; PLB, phospolamban; PMCA, plasmalemma calcium ATPase; PN, periostin; PVA, parvalbumin; RGN, regucalcin; RMP, resting membrane potential; RyR1‐CRC, ryanodine receptor calcium release channel; SERCA; sarcoplasmic reticulum calcium ATPase; SG, sarcoglycan; SLN, sarcolipin; SOCE, store‐operated calcium entry; SRL, sarcalumenin; SSPN, sarcospan; STAC3, SH3 and cysteine‐rich domain 3 protein; STIM1, stromal interaction molecule 1; TN, troponin; TP, tropomyosin; TRDN, triadin; VDAC, voltage‐dependent anion channel

The arrangement of these diverse Ca^2+^‐regulatory proteins in the complex membrane system of skeletal muscle fibres is outlined in the diagram of Figure 3. High cytosolic Ca^2+^‐levels result in TnC‐mediated alterations in the troponin complex and its interactions with tropomyosin. This in turn affects tropomyosin conformation and acto‐myosin interaction patterns within the contractile apparatus that allow sarcomeric shortening and muscle filament sliding [94]. Crucial cytosolic Ca^2+^‐buffering is mediated by proteins such as regucalcin and parvalbumin [95], which thereby play a key role in Ca^2+^‐dependent uptake processes of this critical second messenger [51]. The swift re‐uptake of Ca^2+^‐ions into the lumen of the sarcoplasmic reticulum is provided by slow and fast isoforms of the SERCA‐type Ca^2+^‐ATPases, in conjunction with their regulatory subunits, that is, slow‐type phospholamban and fast‐type sarcolipin, which induces skeletal muscle relaxation [96].

## 4 CALCIUM HYPOTHESIS OF MUSCULAR DYSTROPHY

The experimental evidence that L‐type Ca^2+^‐channel (Ca_v_) function may be linked to expression levels of dystrophin in skeletal muscle tissue [55] suggests a potential operative connection between the physiological mechanisms of excitation–contraction coupling and the stabilising and signalling role of the dystrophin‐glycoprotein complex [10]. However, it has not yet been established whether the lack of dystrophin in X‐linked muscular dystrophy only indirectly modifies excitation–contraction coupling by causing sarcolemmal disruptions or directly affects changes in Ca^2+^‐regulation and cellular signalling. Besides the dihydropyridine receptor, additional molecular interaction patterns between the dystrophin complex and ion channels may include transient receptor potential channels TRPC1‐4 and the voltage‐dependent skeletal muscle Na^+^‐channel Na_v_1.4, as reviewed by Leyva‐Leyva et al. [97].

**FIGURE 4:**
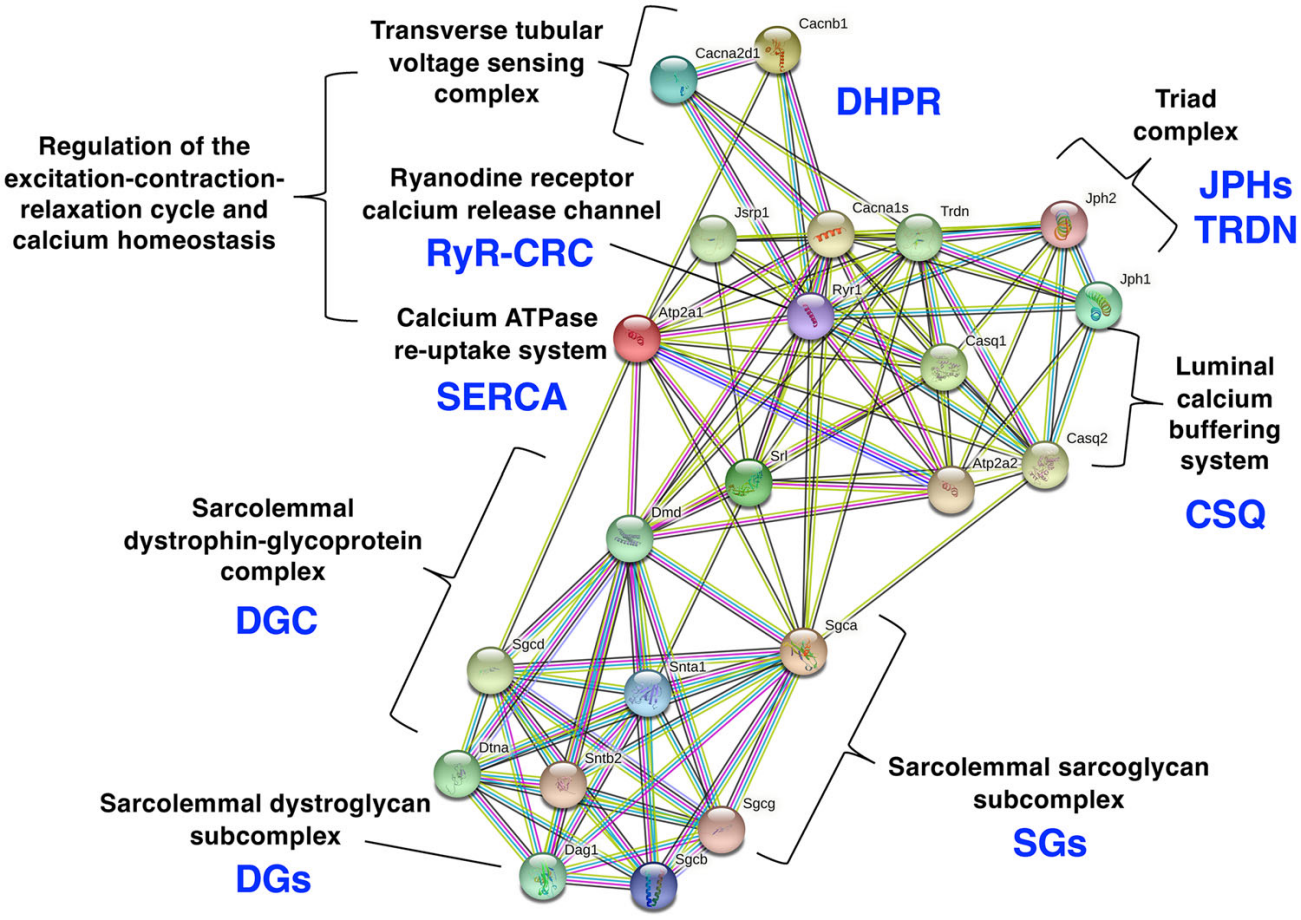
Potential protein interactions between the sarcolemmal dystrophin‐glycoprotein complex and proteins involved in the regulation of excitation–contraction coupling using bioinformatic STRING analysis [98] of proteins identified by the mass spectrometric survey of skeletal muscle membrane fractions [21, 99]. The various evidence types that contribute to STRING are discernible in the visual networks by lines of different colours, including neighbourhood evidence, co‐occurrence evidence, experimental evidence, database evidence and co‐expression evidence [98]. In our analysis, a ‘high confidence’ threshold (score of 0.7) was used to generate the STRING network. CSQ, calsequestrin; DGC, dystrophin‐glycoprotein complex; DGs, dystroglycans; JPHs, junctophilins; RyR‐CRC, ryanodine receptor calcium release channel; SERCA, sarcoplasmic reticulum calcium ATPase; SGs, sarcoglycans; TRDN, triadin

Figure 4 displays the findings of the bioinformatic STRING analysis [98] of key proteins of Ca^2+^‐handling and the core members of the dystrophin complex, identified by the mass spectrometric analysis of enriched skeletal muscle membrane fractions [99]. The protein hub consisting of the transverse tubular voltage‐sensor, the triadic Ca^2+^‐release units and the luminal Ca^2+^‐buffering system exhibits potential linkages to the full‐length Dp427‐M isoform of dystrophin and its associated sarcolemmal complex. Thus, in the absence of the protective dystrophin lattice and resulting destabilisation of the crucial trans‐plasmalemmal actin–dystroglycan–laminin axis at the skeletal muscle periphery, membrane rupturing can be induced by the enormous load bearing and continuous mechanical strains caused by repeated excitation–contraction–relaxation cycles [27, 57, 58] and thereby trigger dysregulation of Ca^2+^‐homeostasis [33, 34, 66, 100].

The calcium hypothesis of dystrophinopathy assumes that abnormal Ca^2+^‐handling and impaired cellular signalling events at the level of the sarcolemma, transverse tubules, triad junctions, the luminal sarcoplasmic reticulum and the sarcosol are responsible for increased Ca^2+^‐dependent muscle protein degradation in dystrophin‐deficient skeletal muscle fibres [38]. The dysregulation of Ca^2+^‐signalling and loss of sarcolemmal function [101] is related to rendering muscle fibres more susceptible to necrosis causing contractile weakness. Hyper‐sensitivity to eccentric contraction‐induced loss of strength is closely linked to electrophysiological dysfunction of the sarcolemma membrane. Importantly, a recent study by Baumann et al. [102] suggests that the contractile weakness in injured muscles from the *mdx‐23* model of Duchenne muscular dystrophy is most likely not associated with just one pathophysiological mechanism, but rather a series of steps that disrupt the process of skeletal muscle contraction. Hence, the molecular pathogenesis of dystrophinopathy appears to be highly complex and involves a multifaceted interplay of dysregulated processes. The chronic loss of contractile fibres and damage to cyto‐architectural integrity triggers mitochondrial dysfunction, fat substitution, reactive myofibrosis and the recruitment of macrophages and other immune cells causing sterile muscle inflammation [56, 103, 104]. Micro‐rupturing of the dystrophin‐lacking surface membrane causes the introduction of Ca^2+^‐leak channels [36, 38, 105], such as transient receptor potential channel isoforms TRPC1 and TRPC3 [37, 106], and the resulting elevation of cytosolic Ca^2+^‐levels was shown to be associated with increased levels of proteolytic degradation of skeletal muscle proteins [36, 37, 38]. Additional important aspects of the cellular pathogenesis of dystrophinopathy are the occurrence of stretch‐induced damage during eccentric contractions [29, 30, 31] and mitochondrial abnormalities involving the mitochondrial transition pore and the production of reactive oxygen by the enzyme NADPH oxidase [46, 47, 48].

Thus, the enhanced activity of Ca^2+^‐activated proteases, such as calpains, is directly involved in fibre destruction [40, 107], besides the secondary disturbance of skeletal muscle function by the abnormal regulation of the Ca^2+^‐related excitation–contraction coupling mechanism and muscle relaxation [39, 40, 41]. This is reflected by significant alterations in the expression of a variety of proteins involved in excitation–contraction coupling [108, 109, 110] and store‐operated Ca^2+^‐entry [111, 112, 113] in X‐linked muscular dystrophy. Of note, dystrophin‐lacking skeletal muscles are characterised by significantly impaired Ca^2+^‐release from the sarcoplasmic reticulum in muscular dystrophy [43, 44, 45, 114] and the dystrophic phenotype of abnormal excitation–contraction coupling is exacerbated in dystrophin/utrophin double‐KO fibres [115]. Interestingly, the overexpression of insulin‐like growth factor IGF‐I improves excitation–contraction coupling in dystrophic skeletal muscle by the modification of charge movements and intracellular Ca^2+^‐transients, possibly by a protein kinase PKC‐associated process that affects the selective ion conductance of the transverse tubular L‐type Ca^2+^‐channel [116].

## 5 MUSCLE PROTEOMICS AND PROFILING OF ABNORMAL CALCIUM HANDLING IN DYSTROPHINOPATHY

Skeletal muscle proteomics has made enormous technical advances over the last two decades and has resulted in the mass spectrometric identification of over 10,000 muscle‐associated proteoforms [117]. Large‐scale proteomic studies have especially focussed on the systematic profiling of normal human skeletal muscle specimens (*vastus lateralis*, various diagnostic biopsy specimens) [118, 119, 120, 121] and the comparison of human fibre types (*vastus lateralis, trapezius* and *deltoideus*) [122, 123, 124, 125], as well as the characterisation of wild type mouse skeletal muscle (*gastrocnemius*, leg muscle tissue, diaphragm) [21, 126, 127, 128], the comparison of rodent fibre types (*gastrocnemius, soleus, tibialis anterior* and *extensor digitorum longus*) [129, 130, 131, 132, 133] and single muscle fibre analyses of mouse muscles (*soleus* and *extensor digitorum longus*) [134, 135, 136]. These comprehensive cataloguing studies have clearly confirmed the presence of diverse protein constituents in the skeletal muscle proteome that are involved in the regulation of excitation–contraction coupling, the maintenance of triad structure and the mediation of spatial and temporal changes in Ca^2+^‐related signalling events. Table 1 lists the proteomic identification of the above‐described key players involved in the regulation of excitation–contraction coupling, muscle relaxation and Ca^2+^‐homeostasis, including muscle proteins located in the sarcolemma (ATP2B1, ATP2B4), transverse tubules (CACNA1S, CACNA2D1, CACNB1), junctional sarcoplasmic reticulum (RYR1, TRDN, JPH1, JPH2, JSRP1, STAC3, STIM1), terminal cisternae (CASQ1, CASQ2) and longitudinal tubules of the sarcoplasmic reticulum (ATP2A1, ATP2A2, SRL) [21, 99].

**TABLE 1:** Proteomic profiling of key components involved in the regulation of the excitation–contraction–relaxation cycle and calcium homeostasis in skeletal muscle

Accession	Protein name	Gene name	Coverage (%)	Unique peptides	Molecular mass (kDa)
E9PZQ0	Ryanodine receptor 1	RYR1	57.52	275	564.7
Q02789	Voltage‐dependent L‐type calcium channel, alpha‐1S	CACNA1S	14.62	26	211.4
O08532	Voltage‐dependent calcium channel, alpha‐2/delta‐1	CACNA2D1	49.95	53	124.6
Q8R3Z5	Voltage‐dependent L‐type calcium channel, beta‐1	CACNB1	53.83	29	57.7
O09165	Calsequestrin‐1	CASQ1	52.10	20	46.3
O09161	Calsequestrin‐2	CASQ2	26.02	9	48.1
Q7TQ48	Sarcalumenin	SRL	46.5	52	99.1
E9Q9K5	Triadin	TRDN	16.59	9	77.8
Q9ET80	Junctophilin‐1	JPH1	11.06	7	71.9
Q9ET78	Junctophilin‐2	JPH2	18.82	9	74.6
Q3MI48	Junctional sarcoplasmic reticulum protein 1	JSRP1	53.01	16	36.1
Q8BZ71	SH3 and cysteine‐rich domain‐containing protein STAC3	STAC3	3.06	1	41.0
P70302	Stromal interaction molecule 1	STIM1	25.40	18	77.5
Q8R429	Calcium ATPase SERCA1	ATP2A1	66.10	87	109.4
O55143‐2	Calcium ATPase SERCA2	ATP2A2	42.08	21	109.7
G5E829	Plasma membrane calcium‐transporting ATPase 1	ATP2B1	21.15	13	134.7
Q6Q477‐2	Plasma membrane calcium‐transporting ATPase 4	ATP2B4	10.12	4	122.2

Based on the knowledge generated by proteomic cataloguing studies, a considerable number of comparative and mass spectrometry‐based investigations have determined proteome‐wide changes in various types of dystrophic skeletal muscle specimens using both restricted amounts of Duchenne/Becker's patient biopsy material [22, 137, 138, 139] and especially a large variety of spontaneous or bio‐engineered animal models including dystrophic mice, pigs and dogs [21, 23, 99, 127, 140, 141, 142, 143, 144, 145, 146, 147, 148, 149, 150, 151, 152, 153, 154, 155, 156, 157, 158, 159, 160, 161, 162]. General listings of proteomic biomarkers of dystrophinopathy, covering both muscle tissue and biofluids such as serum, have been published in extensive reviews [11, 12, 13, 14, 15, 24, 56]. Proteome‐wide changes due to dystrophin deficiency include proteins involved in skeletal muscle contraction, regulation of excitation–contraction coupling, cellular signalling, bioenergetic pathways, metabolite transportation, maintenance of the cytoskeletal networks, cyto‐architectural integrity, modulations of the extracellular matrix and the cellular stress response. Comparative surveys of dystrophic fibres included proteomic studies with special focus on changes in Ca^2+^‐regulatory proteins due to abnormal dystrophin expression [160, 161, 162]. The combined results of the various mass spectrometric investigations into the molecular pathogenesis of dystrophinopathy indicate that the drastic decrease in Dp427‐M and its associated glycoproteins is accompanied by significantly reduced expression levels of key proteins of excitation–contraction coupling, including junctophilin‐1, junctophilin‐2, calsequestrin, sarcalumenin, the ryanodine receptor Ca^2+^‐release channel of the triad junction and the α1S/α2/δ/β‐dihydropyridine receptor subunits of the L‐type Ca^2+^‐channel complex of the transverse tubules, which agrees with previous biochemical and physiological investigations into the fate of Ca^2+^‐regulatory proteins in X‐linked muscular dystrophy [108, 109].

A general bioanalytical issue with proteomic studies that use crude extracts of skeletal muscle tissue samples as starting material is the heterogeneity of these specimens. Animal muscle samples and human muscle biopsies usually contain a variety of different muscle fibre types, including myocytes with greatly divergent metabolic and contractile profiles, as well as various subtypes of fibroblasts, adipocytes, satellite cells, immune cells, motor neurons, capillaries and biofluids. During neuromuscular disease processes, the ratio of these cells types can drastically vary based on complex processes such as selective patterns of myonecrosis, reactive myofibrosis, fat substitution, inflammation and fibre type shifting [83]. Fast‐twitching muscle fibres were shown to be preferentially affected in Duchenne muscular dystrophy [163]. Thus, the complexity of cell types within muscle tissues and their dynamic character may complicate the interpretation of mass spectrometry‐based proteomic surveys of crude protein extracts. It is therefore possible that the proteomic identification of changes in distinct proteins is partially linked to disease‐related changes in fibre type ratios and/or the replacement of contractile fibres with fibroblasts or adipocytes. To evaluate this issue, independent verification studies can be carried out with standardised cell biological methods, such as immunofluorescence microscopy [6]. These additional analyses of individual muscle proteins can then be used to substantiate altered expression patterns as determined by biochemical investigations. For example, the reduced expression of the luminal Ca^2+^‐binding protein sarcalumenin in dystrophic muscles, as detected by two‐dimensional gel electrophoresis and immunoblotting, was independently verified by immunofluorescence microscopy [109]. Confocal microscopy with highly specific antibodies to proteins of interest can therefore help to corroborate changes in distinct ion‐regulatory proteins at the level of individual muscle fibres. Verification studies can determine whether protein expression changes are specific to myocytes or are a reflection of overall alterations in general cell composition and/or muscle fibre type shifting.

The results summarised in Table 2 list findings from major proteomic studies that have established alterations in the expression levels of proteins involved in the regulation of the excitation–contraction–relaxation cycle and Ca^2+^‐homeostasis in dystrophic skeletal muscles. The table gives an overview of individual proteoforms identified by mass spectrometry, the various bioanalytical approaches taken for comparative analyses and the specific skeletal muscle tissues that were studied. The technical approaches for large‐scale protein separation included subcellular fractionation [54, 156], membrane agglutination [99], isoelectric focussing [157], one‐dimensional gel electrophoresis [153, 154, 156], two‐dimensional gel electrophoresis [22, 142, 146, 148, 160, 161, 162] and reversed phase liquid chromatography [22, 99, 139, 143, 145, 146, 147, 148, 149, 150, 151, 152, 153, 154, 155, 156, 157, 158, 159], as well as chemical crosslinking [156]. Protein digestion protocols involved in‐gel, on‐membrane or in‐solution treatment with trypsin plus other types of proteases. Labelling protocols encompassed differential fluorescent tagging prior to gel electrophoretic separation using difference gel electrophoresis (2D‐DIGE) [22, 142, 146, 148, 162], stable isotope labelling by amino acids in cell culture (SILAC) [147], isotope‐coded protein labelling (ICPL) [153] and tandem mass tagging (TMT) [145, 157]. Multidimensional protein identification technology (MudPIT) [145], liquid chromatography tandem mass spectrometry (LC‐MS/MS) [22, 99, 139, 143, 145, 146, 147, 148, 149, 150, 151, 152, 153, 154, 155, 156, 157, 158, 159] and matrix assisted laser desorption/ionisation time‐of‐flight mass spectrometry (MALDI‐ToF) [142, 160, 161, 162] were applied for routine protein identification. The identified changes in Ca^2+^‐handling proteins using top‐down/gel‐based versus bottom‐up/gel‐free approaches were found to be mostly complementary and confirmatory in nature.

**TABLE 2:** Mass spectrometry‐based proteomic profiling of tissue‐associated proteins involved in the regulation of the excitation–contraction–relaxation cycle and Ca^2+^‐homeostasis in dystrophic skeletal muscles

Species/specimens	Skeletal muscle tissue	Identified proteoforms	Bioanalytical approaches	References
Human skeletal muscle biopsies				
Duchenne and Becker's muscular dystrophy biopsies	*Vastus lateralis*	Decreased junctophilin‐1	2D‐DIGE, LC‐MS/MS	Capitanio et al. [22]
Becker's muscular dystrophy biopsies	*Vastus lateralis*	Decreased fast SERCA1 Ca^2+^‐ATPase	LC‐MS/MS	Capitanio et al. [139]
Mouse leg muscles				
Dystrophic *mdx‐23* mouse	Hind limb muscle extracts	Decreased fast calsequestrin CSQ1 and sarcalumenin	2D‐GE, MALDI‐ToF MS, 2D Stains‐All	Doran et al. [160]
Dystrophic *mdx‐23* mouse	*Soleus, extensor digitorum longus, flexor digitorum brevis* and *interosseus*	Decreased parvalbumin	2D‐DIGE, LC‐MS/MS	Carberry et al. [148]
Dystrophic *mdx‐4cv* mouse	Membrane fractions from hind limb muscle extracts	Decreased fast calsequestrin CSQ‐1, sarcalumenin, and L‐type Ca^2+^‐channel beta‐1	Lectin affinity purification, LC‐MS/MS	Murphy et al. [99]
Dystrophic *mdx‐4cv* mouse	*Soleus, extensor digitorum longus, flexor digitorum brevis* and *interosseus*	Decreased parvalbumin	LC‐MS/MS	Holland et al. [151]
Dystrophic *mdx‐4cv* mouse	Microsomal fraction from hind limb muscle extracts	Decreased parvalbumin	LC‐MS/MS, IB	Murphy et al. [150]
Dystrophic *mdx‐23* mouse	*Quadriceps femoris*	Decreased fast SERCA1 Ca^2+^‐ATPase	Ultracentrifugation, SDS‐PAGE, on‐membrane digestion, LC‐MS/MS	Murphy et al. [154]
Dystrophic *mdx‐4cv* mouse	Hind limb muscle microsomes following chemical XL	Increased tendency of sarcalumenin oligomerisation	XL, SDS‐PAGE, LC‐MS/MS	Murphy et al. [156]
Dystrophic *mdx‐52* mouse	*Tibialis anterior*	Decreased fast SERCA1 Ca^2+^‐ATPase	TMT, IEF, LC‐MS/MS	Van Westering et al. [157]
Mouse diaphragm muscle				
Dystrophic *mdx‐23* mouse	Diaphragm	Decreased regucalcin	2D‐GE, MALDI‐ToF MS, 2D‐IB	Doran et al. [161]
Dystrophic *mdx‐23* mouse	Diaphragm	Decreased regucalcin	2D‐DIGE, MALDI‐ToF MS	Doran et al. [142]
Dystrophic *mdx‐23* mouse	Diaphragm, as compared to extraocular muscle	Decreased fast calsequestrin CSQ1	TMT isobaric mass tagging, MudPIT, LC‐MS/MS	Matsumura et al. [145]
Dystrophic *mdx‐23* mouse	Diaphragm, as compared to various hind limb muscles	Decreased fast calsequestrin CSQ1, parvalbumin and troponin TnC	2D‐DIGE, LC‐MS/MS	Carberry et al. [148]
Dystrophic *mdx‐4cv* mouse	Diaphragm	Decreased parvalbumin	LC‐MS/MS	Holland et al. [149]
Dystrophic *mdx‐4cv* mouse	Diaphragm, as compared to various hind limb muscles	Decreased parvalbumin	LC‐MS/MS	Holland et al. [151]
Dystrophic *mdx‐23* mouse following experimental exon‐skipping therapy	Diaphragm	Decreased regucalcin; reversal of expression changes in fast calsequestrin CSQ1	2D‐DIGE, MALDI‐ToF, 2D‐IB	Doran et al. [162]
Dystrophic *mdx‐4cv* mouse	Diaphragm	Decreased parvalbumin, junctophilin‐1 and junctophilin‐2	LC‐MS/MS	Murphy et al. [21]
Silac mouse				
Dystrophic *mdx‐23* mouse	*Gastrocnemius*	Decreased parvalbumin	SILAC mouse analysis, LC‐MS/MS	Rayavarapu et al. [147]
Dog muscles				
Dystrophic GRMD dog	*Vastus lateralis*	Decreased slow SERCA2 Ca^2+^‐ATPase	ICAT, LC‐MS/MS	Guevel et al. [143]
Dystrophic GRMD dog	*Biceps femoris* following allogenic MuStem cell application	MuStem cell‐induced decrease in junctophilin‐1 and L‐type Ca^2+^‐channel alpha‐2/delta	ICPL, SDS‐PAGE, LC‐MS/MS	Lardenois et al. [153]
Pig muscles				
Dystrophic DMD pig	*Biceps femoris*	Decreased fast troponin TnC	LC‐MS/MS	Fröhlich et al. [152]

Listed are comparative and mass spectrometry‐based proteomic investigations with an analytical focus on the identification and characterisation of key Ca^2+^‐regulatory proteins. More comprehensive listings that discuss a wider range of both tissue‐ and biofluid‐associated proteoforms with an altered expression in X‐linked muscular dystrophy have previously been published [11, 12, 13, 14, 15]. 2D, two‐dimensional; DIGE, two‐dimensional difference gel electrophoresis; GE, gel electrophoresis; ICAT, isotope‐coded affinity‐tag based protein profiling; ICPL, isotope‐coded protein labelling; IEF, isoelectric focussing; IB, immunoblotting; LC‐MS/MS, liquid chromatography tandem mass spectrometry; MALDI‐ToF, matrix assisted laser desorption/ionisation time‐of‐flight; MudPIT, multidimensional protein identification technology; SDS‐PAGE, sodium dodecyl sulphate polyacrylamide gel electrophoresis; SILAC, stable isotope labelling by amino acids in cell culture; Stains‐All, cationic carbocyanine dye staining; TMT, tandem mass tag; XL, chemical crosslinking.

Decreased expression of the triad component junctophilin was shown to occur in both biopsy specimens from the *vastus lateralis* muscle of patients afflicted with Duchenne muscular dystrophy [22] and skeletal muscles from dystrophic animal models, such as the GRMD dog *biceps femoris* muscle [153] and the *mdx‐4cv* mouse diaphragm [21]. Since junctophilins maintain the structural integrity within the contact sites of the triad junction between the transverse tubules and the sarcoplasmic reticulum [85, 87], their reduced expression probably negatively affects the proper physiological coupling between the voltage‐sensing L‐type channel and the ryanodine receptor Ca^2+^‐release channel [32, 33, 34]. In analogy, the α1S/α2/δ/β‐dihydropyridine receptor subunits of the L‐type Ca^2+^‐channel complex of the transverse tubules and the junctional ryanodine receptor Ca^2+^‐release channel are also reduced in dystrophinopathy as judged by the proteomic profiling of the dystrophic and highly fibrotic *mdx‐4cv* diaphragm muscle [21]. These alterations might play a major cyto‐architectural and functional role in the impaired excitation–contraction mechanism in dystrophin‐deficient skeletal muscle fibres [33].

Both, luminal and cytosolic Ca^2+^‐buffering are essential for the temporal and spatial regulation of Ca^2+^‐homeostasis [95]. Thus, the proteomic observations of drastically reduced levels of calsequestrin and sarcalumenin in the lumen of the sarcoplasmic reticulum [99, 144, 146, 160, 162], and regucalcin and parvalbumin in the sarcosol [21, 142, 146, 147, 148, 149, 150, 151, 160, 162] of dystrophic skeletal muscles indicate drastically impaired Ca^2+^‐binding patterns in dystrophinopathy. This would cause both reduced levels of Ca^2+^‐ions being available for the passive release process during excitation–contraction coupling and altered patterns of Ca^2+^‐dependent signalling in the cytosol [32, 33, 34]. In addition, the proteomic identification of lower abundances of SERCA‐type Ca^2+^‐ATPases in dystrophic fibres suggests also impaired Ca^2+^‐reuptake into the lumen of the sarcoplasmic reticulum during muscle relaxation [139, 143, 154, 157]. Hence, systematic mass spectrometric surveys of dystrophic skeletal muscles have clearly established significant changes in key proteins involved in the regulation of the excitation–contraction–relaxation cycle. The promising developments in single cell proteomics in the field of basic and applied myology [124] will hopefully also be soon applied to the detailed characterisation of the Ca^2+^‐regulatory apparatus in single cellular entities from heterogeneous dystrophic fibre populations.

**FIGURE 5:**
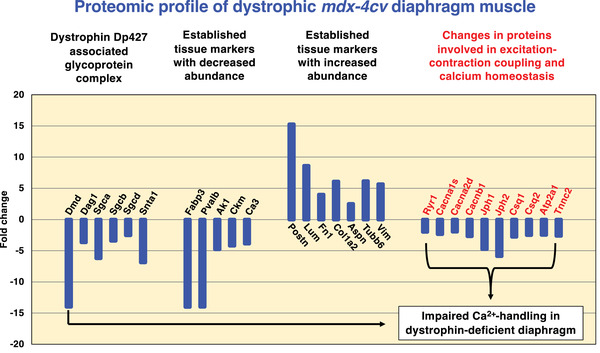
Overview of key proteomic changes in the dystrophic *mdx‐4cv* diaphragm. Shown are abundance changes in the form of bar diagrams illustrating the loss of core members of the dystrophin‐glycoprotein complex (Dmd, dystrophin; Dag1, α/β‐dystroglycan; Sgca, α‐sarcoglycan; Sgb, β‐sarcoglycan; Sgcd, δ‐sarcoglycan; Snta1, α1‐syntrophin) and concomitant reductions in established proteomic tissue markers (Fabp3, fatty acid binding protein 3; Pvalb, parvalbumin; Ak1, adenylate kinase; Ckm, creatine kinase; Ca3, carbonic anhydrase), myofibrosis‐related increases in extracellular matrix proteins (Postn, periostin; Lum, lumican; Fn1, fibronectin; Col1a2, collagen; Asp, asporin), the apparent compensatory upregulation of cytoskeletal components (Tubb6, tubulin; Vim, vimentin) and decreased expression of crucial Ca^2+^‐handling proteins (Cacna1s, α1S‐dihydropyridine receptor; Cacna2d, α2/δ‐dihydropyridine receptor; Cacnb1, β1‐dihydropyridine receptor, RyR1, ryanodine receptor isoform 1 Ca^2+^‐release channel; Jph1, junctophilin‐1; Jph2, junctophilin‐2; Csq1, fast calsequestrin 1; slow calsequestrin 2; Csq2); Atp2a1, fast sarcoplasmic reticulum Ca^2+^‐ATPase; Tnnc2, Ca^2+^‐binding troponin subunit TnC) in dystrophin‐lacking fibres. Mass spectrometric analyses were carried out as previously described in detail [21]

Figure 5 summarises representative findings from the proteomic profiling of the dystrophic diaphragm from the genetic *mdx‐4cv* mouse model of Duchenne muscular dystrophy [21] demonstrating the reduced expression of Ca^2+^‐handling proteins in dystrophin‐lacking fibres. The mutant status of the *mdx‐4cv* diaphragm is confirmed by the drastic reduction in dystrophin isoform Dp427‐M (Dmd) and associated decrease in α/β‐dystroglycan (Dag1), α/β/δ‐sarcoglycans (Sgca, Sgb, Sgcd) and α1‐syntrophin (Snta1). The illustration also displays the fold‐change in abundance of well‐established decreased versus increased markers of dystrophinopathy. This includes significantly lower levels of fatty acid binding protein (Fabp3), parvalbumin (Pvalb), adenylate kinase (Ak1), creatine kinase (Ckm) and carbonic anhydrase (Ca3) in dystrophic muscle tissue [141, 164, 165], as compared to an increased abundance of these markers in biofluids such as serum, saliva and urine [11, 12, 13, 14]. These alterations in protein expression suggest drastic changes in metabolite transportation, bioenergetic pathways and nucleotide metabolism, as well as the shedding of fatty acid binding protein FABP3, the enzyme creatine kinase and carbonic anhydrase isoform CA3 from leaky and dystrophin‐lacking sarcolemma membranes [15]. Elevated concentrations in skeletal muscle tissues have been established for the extracellular matrix components periostin (Postn), lumican (Lum), fibronectin (Fn1), collagen (Col1a2) and asporin (Asp), which is associated with reactive myofibrosis [21, 56, 148, 149], as well as increased levels of tubulin (Tubb6) and vimentin (Vim) [21, 99, 146, 150] that is indicative of cytoskeletal remodelling in response to dystrophin deficiency.

**FIGURE 6:**
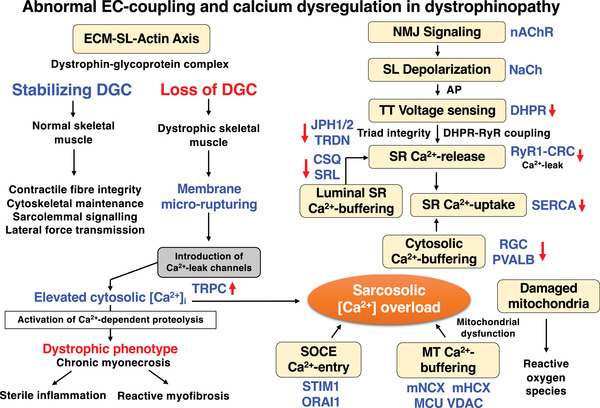
Diagrammatic summary of impaired excitation–contraction coupling and dysregulation of Ca^2+^‐handling in X‐linked muscular dystrophy. The figure gives an overview of the complex changes that are caused by dystrophin deficiency, which introduces membrane micro‐rupturing and Ca^2+^‐leak channels in the sarcolemma. The resulting sarcosolic Ca^2+^‐overload activates Ca^2+^‐dependent proteolysis and fibre destruction, as well as secondary alterations due to reactive myofibrosis and chronic sterile inflammation. Altered Ca^2+^‐levels are associated with disturbed cellular signalling cascades and negatively affect excitation–contraction coupling and Ca^2+^‐homeostasis, including impaired voltage‐sensing, altered Ca^2+^‐release, diminished triad maintenance, lower levels of both cytosolic and luminal Ca^2+^‐buffering, as well as dysregulated Ca^2+^‐uptake. Store‐operated Ca^2+^‐entry and damaged mitochondria and the production of reactive oxygen species are also characteristic features of the dystrophic phenotype. AP, action potential; CSQ, calsequestrin; DGC, dystrophin‐glycoprotein complex; DHPR, dihydropyridine receptor; EC, excitation contraction; ECM, extracellular matrix; JPH, junctophilin; MCU, mitochondrial calcium uniporter; mHCX, mitochondrial hydrogen‐calcium exchanger; mNCX, mitochondrial sodium‐calcium exchanger; MT, mitochondria; ORAI1, calcium release‐activated calcium modulator 1; NaCh, sodium channel; nAChR, nicotinic acetylcholine receptor; NMJ, neuromuscular junction; PVALB, parvalbumin; RGN, regucalcin; SERCA, sarcoplasmic reticulum calcium ATPase; SL, sarcolemma; SR, sarcoplasmic reticulum; SRL, sarcalumenin; STIM1, stromal interaction molecule 1; TRDN, triadin; TRPC, transient receptor potential channel; TT, transverse tubules; VDAC, voltage dependent anion channel

The diagram displayed in Figure 6 summarises the impaired excitation–contraction coupling process and abnormal Ca^2+^‐handling in X‐linked muscular dystrophy. Loss of the dystrophin‐glycoprotein complex [35] renders the sarcolemma more susceptible to membrane micro‐rupturing [25, 26, 27], which in turn triggers the introduction of Ca^2+^‐leak channels that cause sarcosolic Ca^2+^‐overload [36, 37, 38, 106]. The activation of Ca^2+^‐dependent proteolytic enzymes is of central importance for fibre degeneration [107]. Proteolysis‐related tissue necrosis is then followed by reactive myofibrosis that results in loss of muscle elasticity [56, 149]. Abnormal cytosolic Ca^2+^‐levels are closely linked to impaired cellular signalling mechanisms [32, 33, 34]. The flow chart lists clearly identified changes in proteins involved in excitation–contraction coupling and Ca^2+^‐homeostasis based on proteomic, biochemical, physiological and cell biological studies. Representative changes in Ca^2+^‐regulatory proteins as determined by mass spectrometry‐based proteomics are listed in Table 2 and Figure 5. Following neuromuscular transmission at the neuromuscular junction and the triggering of membrane depolarisation via the transient opening of the nicotinic acetylcholine receptor channel, action potentials are propagated along the muscle surface membrane with the help of voltage‐dependent Na^+^‐channels. Mass spectrometric studies indicate that the subsequent signalling steps of excitation–contraction coupling are affected by reduced expression levels of key Ca^2+^‐handling proteins.

This includes (i) the dihydropyridine receptor L‐type Ca^2+^‐channel complex (Cacna1s, Cacna2d, Cacnb1) that is involved in signal transduction at the transverse tubules, (ii) the tetrameric ryanodine receptor Ca^2+^‐release channel complex (RyR1) of the junctional sarcoplasmic reticulum, (iii) the crucial junctional components triadin (Trdn) and junctophilins (Jph1, Jph2) that play a key role in triad junction maintenance, (iv) the essential luminal Ca^2+^‐buffering proteins calsequestrin (Csq1, Csq2) located in the terminal cisternae region of the sarcoplasmic reticulum and sarcalumenin (Srl) in the longitudinal tubules, (v) the cytosolic Ca^2+^‐binding proteins regucalcin (Rgn) and parvalbumin (Pvalb) and (vi) the SERCA‐type Ca^2+^‐pumps (Atp2a1) that are responsible for the efficient Ca^2+^‐reuptake during muscle relaxation. These proteomic changes are probably associated with impaired voltage‐sensing, altered Ca^2+^‐release, diminished triad maintenance, lower levels of both cytosolic and luminal Ca^2+^‐buffering, as well as dysregulated Ca^2+^‐uptake, which ultimately affects the troponin subunit TnC (Tnnc2) mediated linkage to the sarcomeric and Ca^2+^‐dependent control of actin–myosin interactions within the contractile apparatus. Thus, abnormal excitation–contraction coupling appears to play a key role in the pathophysiology of X‐linked muscular dystrophy. In addition, store‐operated Ca^2+^‐entry and abnormal mitochondrial functioning affects the dystrophic phenotype and damaged mitochondria produce increased levels of reactive oxygen species [66, 92, 93].

Besides chronic fibre degeneration and resulting fibrotic changes, sterile inflammation also plays a key role in dystrophinopathy including the migration of monocytes and maturation of M1 and M2 macrophages, as well as the accumulation of CD4^+^ T‐cells, CD8^+^ T‐cells, eosinophils and natural killer cells in dystrophic fibres [104]. Hence, the chronic inflammatory phenotype of dystrophinopathy has to be taken into account [166, 167] together with the occurrence of abnormal ion homeostasis and reactive myofibrosis [56, 149] to develop a more comprehensive understanding of the complex cellular pathogenesis involved in X‐linked muscular dystrophy.

## 6 CONCLUSIONS

Duchenne muscular dystrophy is a highly complex genetic disease that is characterised by primary skeletal muscle wasting, accompanied by respiratory failure and late‐onset cardiomyopathy, as well as various body‐wide disturbances. Basic biomedical studies on muscular dystrophy have used both genetic animal models and patient biopsy specimens to elucidate the pathophysiological mechanisms that underlie contractile weakness, secondary abnormalities and organ crosstalk in dystrophinpathy. Proteomic, biochemical, cell biological and physiological investigations have provided detailed insights into damage pathways involved in changed membrane permeability and abnormal Ca^2+^‐handling in association with deficiency in dystrophin. This has established the biomedical idea that the molecular and cellular pathogenesis of X‐linked muscular dystrophy is based on both changes/adaptations in the sarcolemma and various other cellular systems. Key features of abnormal functioning include dysregulation of ion homeostasis, disturbed cellular signalling, impaired bioenergetic pathways, metabolic disturbances, oxidative stress, sterile inflammation, weakened regenerative capacity, fat substitution and reactive myofibrosis. Current research aims at proteomic biomarkers that can be useful to differentiate between X‐linked muscular dystrophy and other types of muscular dystrophies and common muscle wasting disorders [168]. In this respect, the proteomic changes in essential Ca^2+^‐handling proteins reviewed in this article represent an excellent starting point for the establishment of a novel tissue biomarker signature of abnormal excitation–contraction coupling, impaired muscle relaxation and disturbed Ca^2+^‐regulation due to a specific primary abnormality in an essential skeletal muscle component. However, it is important to emphasise that changes in tissue‐associated proteins, as described in this review, are not ideal for the development of diagnostic assays to be employed for therapy monitoring. The most serious drawback of muscle biopsy procedures is their invasive nature that does not allow repeated sampling over time. Instead, the introduction of ‘liquid biopsy’ approaches using non‐invasive methods would be more suitable for routine testing. Systematic proteomic surveys of biological fluids could be helpful to establish new biomarkers to be used in both basic and applied studies in muscular dystrophy research.

## CONFLICT OF INTEREST

The authors declare no conflict of interest.

## Data Availability

The mass spectrometric raw data used in this review as illustrative examples are available on request.
